# Thyroid Cancer and Fibroblasts

**DOI:** 10.3390/cancers14174172

**Published:** 2022-08-29

**Authors:** Angelica Avagliano, Giuseppe Fiume, Claudio Bellevicine, Giancarlo Troncone, Alessandro Venuta, Vittoria Acampora, Sabrina De Lella, Maria Rosaria Ruocco, Stefania Masone, Nunzio Velotti, Pietro Carotenuto, Massimo Mallardo, Carmen Caiazza, Stefania Montagnani, Alessandro Arcucci

**Affiliations:** 1 Department of Public Health, University of Napoli Federico II, 80131 Naples, Italy; 2 Department of Experimental and Clinical Medicine, University “Magna Graecia” of Catanzaro, 88100 Catanzaro, Italy; 3 Department of Molecular Medicine and Medical Biotechnology, University of Naples Federico II, 80131 Naples, Italy; 4 Department of Clinical Medicine and Surgery, University of Naples Federico II, 80131 Naples, Italy; 5 Department of Advanced Biomedical Sciences, University of Naples Federico II, 80131 Naples, Italy; 6 TIGEM, Telethon Institute of Genetics and Medicine, 80078 Naples, Italy; 7 Medical Genetics, Department of Translational Medical Science, University of Naples Federico II, 80131 Naples, Italy

**Keywords:** thyroid cancer, tumor microenvironment, cancer-associated fibroblasts, thyroid cancer cells

## Abstract

**Simple Summary:**

Thyroid cancer is the most common solid tumor of the endocrine glands. The cancer cell contribution to thyroid cancer development and progression has been studied extensively, whereas the involvement of the tumor microenvironment, particularly of cancer-associated fibroblasts (CAFs), in thyroid cancer growth still must be largely analyzed. The tumor microenvironment, comprising molecules, blood and lymphatic tumor vessels and several non cancer stromal cells, such as CAFs, dramatically influences solid tumor growth and therapy resistance. In particular, investigations on CAF contribution to solid tumor growth and therapeutic resistance represent an important area of oncological research. Moreover, studies focused on the role of CAFs in thyroid cancer could lead to a better comprehension of mechanisms regulating cancer growth and to the development of new therapeutic strategies. Therefore, in this paper, we review the hallmarks of CAFs and their role on thyroid cancer.

**Abstract:**

Thyroid cancer is the most common type of endocrine cancer, and its prevalence continue to rise. Non-metastatic thyroid cancer patients are successfully treated. However, looking for new therapeutic strategies is of great importance for metastatic thyroid cancers that still lead to death. With respect to this, the tumor microenvironment (TME), which plays a key role in tumor progression, should be considered as a new promising therapeutic target to hamper thyroid cancer progression. Indeed, thyroid tumors consist of cancer cells and a heterogeneous and ever-changing niche, represented by the TME, which contributes to establishing most of the features of cancer cells. The TME consists of extracellular matrix (ECM) molecules, soluble factors, metabolites, blood and lymphatic tumor vessels and several stromal cell types that, by interacting with each other and with tumor cells, affect TME remodeling, cancer growth and progression. Among the thyroid TME components, cancer-associated fibroblasts (CAFs) have gained more attention in the last years. Indeed, recent important evidence showed that thyroid CAFs strongly sustain thyroid cancer growth and progression by producing soluble factors and ECM proteins, which, in turn, deeply affect thyroid cancer cell behavior and aggressiveness. Hence, in this article, we describe the thyroid TME, focusing on the desmoplastic stromal reaction, which is a powerful indicator of thyroid cancer progression and an invasive growth pattern. In addition, we discuss the origins and features of the thyroid CAFs, their influence on thyroid cancer growth and progression, their role in remodeling the ECM and their immune-modulating functions. We finally debate therapeutic perspectives targeting CAFs.

## 1. Introduction

The thyroid gland is formed by two lobes connected by the isthmus crossing the midline of the upper trachea at the second and third tracheal rings [1]. The thyroid gland is localized posterior to the sternothyroid and sternohyoid muscles and envelops the cricoid cartilage and tracheal rings. This gland is situated inferior to the laryngeal thyroid cartilage, commonly corresponding to C5-T1 vertebral levels [1]. The functional units of the thyroid gland are represented by the follicles, which differ in size and shape [2]. The major cell type of the follicles is represented by thyrocytes, which form a monolayer epithelium enclosing a central cavity filled with colloid, which is a thyroid hormone store. Each follicle is surrounded by a network of capillaries. Furthermore, neuroendocrine cells, fibroblasts and other stromal cells, such as macrophages, lie close to the follicles or interstitially [2]. The thyroid gland presents the most common lesions associated with surgical pathology practice. In particular, the thyroid lesions include both non-neoplastic and neoplastic pathology [3]. Regarding the neoplastic pathology, it is important to note that thyroid cancer is one of the most common endocrine malignancies, whose incidence has constantly increased worldwide over the past decades [4]. The death rate caused by thyroid cancer is relatively low. However, the rates of disease persistence and recurrence are high and linked to an enhanced incurability, morbidity and mortality of thyroid cancer patients [5]. Thyroid cancer comprises distinct histological types, characterized by different clinical behaviors and aggressiveness [5,6]. In particular, different thyroid cancer types can comprise either indolent tumors with good prognosis and long-term survival, represented by differentiated thyroid cancer (DTC), or more aggressive tumor types, including poorly differentiated thyroid carcinoma (PDTC), anaplastic thyroid cancer (ATC) and medullary thyroid cancer (MTC) [5,6,7,8]. DTC accounts for more than 90% of thyroid tumors and includes papillary thyroid cancer (PTC) and follicular thyroid cancer (FTC) [5,7]. PTC and FTC are typically slowly progressive tumors, displaying a 10-year survival rate of 80–95%. They generally remain localized to the thyroid gland. Distant metastases, ranging from 4% to 15%, predict poor survival, and only 50% of patients with metastatic DTC survive after 10 years [8]. PDTC and ATC are rare cancers and represent 5% and 1% of all thyroid tumors, respectively [5,7]. ATC is a rare thyroid cancer, but it is also the most aggressive tumor type, characterized by pleomorphic histopathological features and rapid progression. For all of these reasons, ATC represents a clinical challenge [6]. DTC, PDTC and ATC arise from the neoplastic transformation of thyroid follicular cells. Differently, MTC, accounting for 5% of thyroid cancer, is derived from the transformation of parafollicular C cells [5,7]. It is noteworthy that the dysregulation of the mitogen-activated protein kinase (MAPK) and phosphatidylinositol-3 kinase (PI3K)/AKT signaling pathways represents the most common driver of thyroid cancer development and progression. In particular, the most common activating mutations in the MAPK pathway are represented by point mutations of BRAF and RAS genes or gene fusions of RET/PTC and TRK. These mutations are fundamental for PTC development. On the other hand, common genetic alterations in the PI3K pathway are considered to be crucial for FTC development, and include mutations in *RAS*, *PIK3CA* and *AKT1*. Thyroid cancer progression and dedifferentiation to PDTC and ATC require a number of additional mutations, such as inactivating mutations in TP53, the activation of the *Wnt*/β-catenin pathway and activating mutations in the promoter. Mutations in the *RET* proto-oncogene are the primary molecular mechanisms underlying MTC tumorigenesis [7]. However, it has been widely demonstrated that the development and progression of tumors depend not only on genetic alterations occurring in cancer cells but also on the bidirectional interactions between tumor cells and their surrounding tumor microenvironment (TME) [9,10,11,12]. In this review, we discuss the recent remarkable progress in understanding the influence of the TME on thyroid cancer growth and progression. In particular, non-cancerous stromal cells of the thyroid TME, mainly represented by cancer-associated fibroblasts (CAFs), are the focus of this review article. Therefore, we describe the origins and features of the thyroid CAFs, their influence on thyroid cancer growth and progression, extracellular matrix (ECM) remodeling and their immune modulating functions. A deep understanding of thyroid-cancer-cell-derived factors and signaling pathways driving thyroid CAF activation and differentiation and CAF-derived soluble and non-soluble factors promoting thyroid cancer growth and progression could be useful to identify new therapeutic strategies targeting the TME and its pro-tumor characteristics.

## 2. Cancer Associated Fibroblasts of Thyroid Desmoplastic Tumor Microenvironment

The TME of solid tumors, such as thyroid cancer, is characterized by an abnormal and ever-changing solid mass, consisting of blood and lymphatic tumor vessels and a dense ECM that contains and stores soluble and insoluble mediators, such as cytokines, growth factors, nutrients, oxygen, adhesion molecules and signaling factors that drive molecular and cellular events affecting tumor development, progression and dissemination [13]. The thyroid TME also consists of a heterogeneous cellular compartment, represented by non-cancerous stromal cell types, such as CAFs, pericytes, immune cells and endothelial cells, that coexist and continuously interact with and support thyroid cancer cells [13,14,15]. The activation of the stromal compartment, also referred to as desmoplastic stroma reaction or desmoplasia, has been widely recognized as an important indicator of cancer progression in many solid tumors [16,17,18]. Regarding thyroid cancers, activated desmoplastic stroma is used as a clinical intraoperative diagnostic marker to characterize thyroid tumor progression and to predict lymph node metastasis [19,20,21,22]. However, despite the recognition of its importance, the desmoplastic stroma reaction is still poorly understood in thyroid cancers and is only gaining more attention and interest in the last few years. Desmoplasia in tumors is characterized by an abundant fibrotic stroma constituted mainly by activated fibroblasts, also called CAFs. CAFs express α-smooth muscle actin (α-SMA), fibroblast activation protein (FAP) and ECM proteins, e.g., tenascin C (Tn-C) [13,17,20]. Several studies demonstrated that the desmoplastic stroma reaction is strongly correlated with an invasive behavior in thyroid tumors. This was confirmed by the study of Harach et al., which identified significant fibrosis in 79% of occult PTCs with an invasive growth pattern. Conversely, slight fibrosis was identified in circumscribed tumors [23]. Koperek et al. demonstrated that the three fibroblast activation markers α-SMA, FAP-α and Tn-C are highly expressed in the peritumoral and intratumoral stromal compartment of MTCs and that the expression of FAP-α and Tn-C correlates with the level of desmoplasia determined by histological analysis [21]. They further demonstrated that the presence of desmoplasia is significantly associated with morphological parameters of invasion and a higher incidence of lymph node metastasis in both PTCs and MTCs [20,21]. On the other hand, the absence of a desmoplastic stroma was shown to be indicative of non-invasive and non-metastasizing thyroid cancers [20]. Cho et al. showed that, among 78 patients with PTC, 65 patients displayed desmoplasia around the tumor mass. CAFs were identified in 42 cases with a desmoplastic stromal reaction. A univariate analysis indicated that both the tumor size and CAFs can represent risk factors for lymph node metastasis. Conversely, a multivariate analysis demonstrated that CAFs were the only independent risk factor for lymph node metastases for PTC patients [24]. Furthermore, Koo’s group demonstrated that the expression of CAF-related proteins in stromal cells and cancer cells of PTC varies according to the histologic subtype and BRAF^V600E^ mutation status and correlates with shorter overall survival [25]. Tarabichi et al. demonstrated that the mutational status of PTCs is associated with the degree of tumor fibrosis, CAF density and CAF activation state [26]. In particular, they focused on the analysis of the desmoplastic stroma reaction in PTCs harboring BRAF^V600E^ mutation (PTCs^BRAFV600E^) [26], which is the most common genetic alteration and an emerging marker of aggressive behavior in PTCs [27,28]. They showed that PTCs^BRAFV600E^ display a higher fibrotic content, increased number of CAFs and enhanced CAF proliferation rate than other PTCs [26]. Finkelstein et al. also confirmed the presence of a higher fibrotic content associated with an increased infiltrative growth in PTCs^BRAFV600E^ than in BRAF^V600E^-negative PTCs [29]. The association between BRAF^V600E^ mutation and an increased CAF number was also reported by the study of Yang et al. [30]. Furthermore, in a mouse model of thyroid cancer, Jolly et al. showed that the activation of BRAF, but not H-RAS, results in a fibrotic response that induces cancer progression and potentially invasion. They also reported that the activation of BRAF, but not H-RAS, increases fibroblast migration and proliferation in vitro, and fibroblast recruitment in vivo [31]. Minna et al. demonstrated that the CAF number is increased at the invasive front of human thyroid cancers, particularly in thyroid cancers harboring BRAF^V600E^ mutation or BRAF-like signaling [32]. Hence, the frequency of desmoplastic-type stroma, the CAF extensive number and more active and proliferative CAFs observed in PTCs^BRAFV600E^ could probably underlie the more aggressive phenotype of this thyroid cancer type [26]. This hypothesis was also supported by the study of Koo‘s group, who reported that PTCs^BRAFV600E^ are associated with a high CAF-related protein expression, which is an established indicator of cancer progression and poor prognosis [25]. The involvement and the critical role of CAFs in contributing to thyroid cancer progression was also validated by other studies, showing that CAFs correlate with the advancement of the pathological N stage of PTC [30]. Considering the presence of distinct CAF subpopulations in the tumor stroma, Weng et al. used three different mRNA-based CAF gene signatures to quantify CAFs in thyroid tumors and to evaluate the correlations of CAFs with the differentiation status and clinicopathological outcomes in patients with thyroid cancers. This study demonstrated that a consistent increase in the three CAF signature scores is correlated with the anaplastic phenotype in dedifferentiation thyroid cancers (DDTCs) and a low thyroid differentiation score in PTCs. Furthermore, it was found that the high CAF scores are positively correlated with invasiveness, increased lymph node metastasis and extrathyroidal extension, a decreased overall survival and, thus, poor clinical outcomes in thyroid cancers. A positive correlation was also found in the high-CAF-score patient group and an increased number of oncogenic signaling pathways, including pathways driving the EMT, apoptosis, TP53, TNF-α/NF-κB, IL2/STAT5 and IL6/JAK/STAT3 signaling pathways [33]. Therefore, an increased desmoplasia and CAF score in thyroid cancers can be useful for clinical practice in predicting cancer aggressiveness and progression and poor clinical outcomes.

Taken together, all of this evidence suggests that the study of the CAF role in thyroid cancer is imperative and fundamental to gaining a better understanding of the molecular and cellular events underlying thyroid cancer development and progression.

## 3. Fibroblasts in Thyroid Cancers: Origin and Features

Human fibroblasts represent a heterogeneous population of differentiated mesenchymal cells of the connective tissue, involved in several physiological and pathological processes, such as tissue homeostasis, ECM turnover, wound healing, fibrosis and cancer [34]. In the human thyroid gland, they are located in the interfollicular connective tissue and represent one of the main constituents of the thyroid stromal tissue [35]. In healthy tissues, as well as in the normal thyroid gland, fibroblasts commonly display a quiescent, non-activated phenotype [31,32,36]. In fact, no α-SMA^+^ fibroblasts are detected in the normal thyroid gland, confirming that activated fibroblasts are commonly absent in physiological conditions of the gland [31,32].

However, during oncogenic insults, fibroblasts are recruited by thyroid cancer cells within the tumor mass [31] and acquire a constitutively activated phenotype [34,37]. Several studies performed on human and murine thyroid tumors demonstrated that the recruitment and infiltration of CAFs into the thyroid TME is pivotal for thyroid cancer progression. By using a PTC murine model of oncogenic BRAF^V600E^ activation, Ryder et al. demonstrated a significant increased infiltration of fibroblasts in thyroid tumors compared with the normal thyroid gland [38]. In the BRAF/PTEN model described by Jolly et al., an increased infiltration of fibroblasts was further observed throughout the tumor mass and along the periphery. Areas enriched with fibroblasts colocalized with areas of increased collagen density. According to this thyroid cancer model, CAFs are recruited at the tumor invasive front, where they modify the surrounding thyroid TME to augment cancer cell migration [31]. According to observations in the BRAF/PTEN murine tumors, Minna et al. demonstrated that α-SMA, collagen and lysyl oxidase colocalize in patient thyroid tumors [32]. In particular, their study revealed the presence of activated fibroblasts localized preferentially along the tumor invasive front, organized in peripheral structures, such as a fibrotic capsule or septa, that are not observed in a non-neoplastic thyroid gland [32].

**Figure 1:**
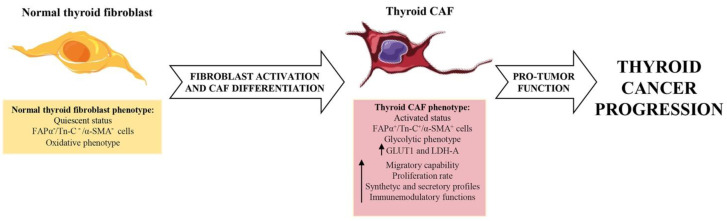
Comparison between normal thyroid fibroblasts and thyroid CAFs. Normal thyroid fibroblasts and thyroid CAFs display molecular and functional differences. Normal thyroid fibroblasts are quiescent cells, characterized by no expression of FAPα, Tn-C and α-SMA markers, which are instead highly expressed by thyroid CAFs. Fibroblast activation and CAF differentiation are associated with CAF metabolic reprogramming towards a more glycolytic phenotype, as demonstrated by the increased expression of GLUT1 and LDH-A. Conversely, normal thyroid fibroblasts prefer oxidative phosphorylation (OXPHOS) for energy supply. Furthermore, thyroid CAFs and their normal counterpart exhibit differences in their migratory capability, proliferation rate and synthetic and secretory profiles. In particular, thyroid CAFs proliferate and migrate faster than normal thyroid fibroblasts and further increase their capability to produce and remodel the ECM, produce inflammatory proteins and modulate immune cells. All of these changes lead thyroid CAFs to acquire a pro-tumorigenic phenotype.

During thyroid cancer development and progression, the bidirectional and continuous interplay between tumor cells and fibroblasts establishes many molecular and functional changes, not only in the tumor compartment but also in the stromal compartment [15]. Indeed, once fibroblasts are accumulated in the thyroid TME, they continue to modify their phenotypic, metabolic and secretory profiles in response to paracrine tumor-derived factors. They gradually lose the molecular and functional characteristics of normal thyroid fibroblasts and acquire a pro-tumorigenic phenotype by differentiating into CAFs (Figure 1) [39,40].

**Figure 2:**
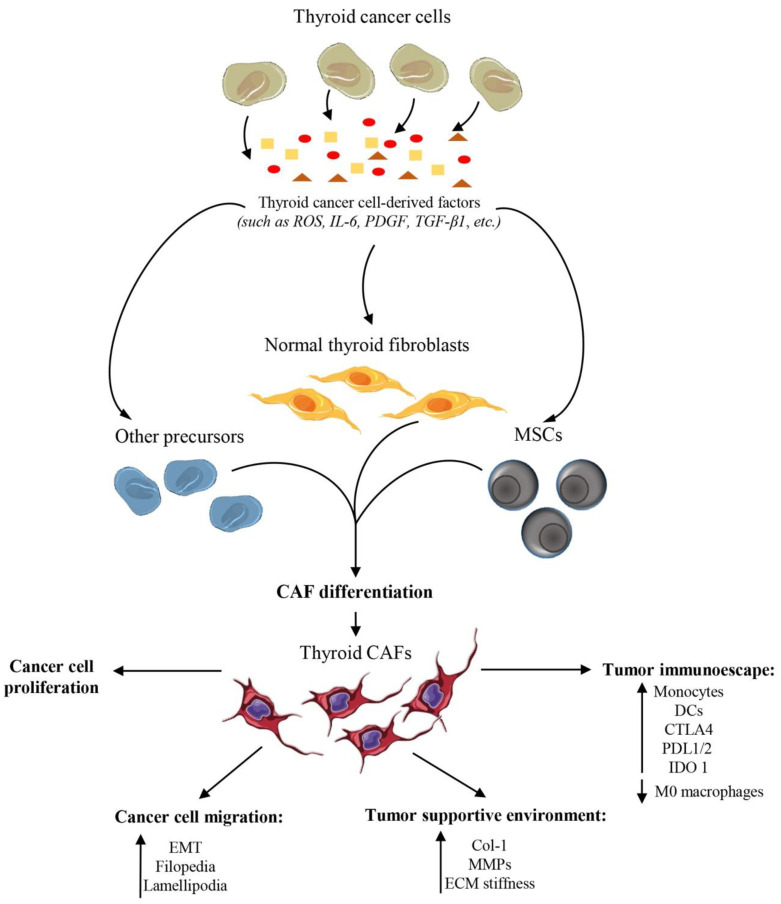
Schematic representation of thyroid cancer cells interacting with CAF precursors. Thyroid cancer cells release many factors in the TME that modify the molecular and functional profile of cells having the potential to differentiate into CAFs. Resident normal thyroid fibroblasts represent the principal source of origin of thyroid CAFs. Thyroid CAFs can also arise from resident MSCs. However, further studies are required to identify other cells of origin of thyroid CAFs. ROS, IL-6, PDGF and TGF-β1 are currently known to be thyroid-cancer-cell-derived factors inducing thyroid CAF activation and differentiation. Further studies are required to identify other factors released by thyroid cancer cells and involved in CAF differentiation. Although CAF-derived factors inducing thyroid cancer progression remain unclarified, it has been demonstrated that thyroid CAFs increase cancer cell proliferation and promote cancer cell migration by driving EMT and enhancing the formation of filopedia and lamellipodia in thyroid cancer cells. Thyroid CAFs further generate a tumor-supportive environment by remodeling the ECM through the expression of proteolytic enzymes, such as MMPs, increasing Col-1 deposition and ECM stiffness. Furthermore, thyroid CAFs promote thyroid growth and progression by modulating the immune cell system and increasing the expression of immune checkpoints, e.g., CTLA4, PDL1/2 and IDO 1.

Hence, resident thyroid fibroblasts, which most probably represent the main source of origin of CAFs in the thyroid TME (Figure 2), differ from thyroid CAFs molecularly and functionally (Figure 1).

It has largely been demonstrated that CAFs from solid tumors, such as breast cancer and melanoma, can originate from distinct cell populations. CAFs can originate from epithelial cells via epithelial-to-mesenchymal transition (EMT), endothelial cells via endothelial-to-mesenchymal transition (EndMT), adipocytes via trans-differentiation and other precursors, such as pericytes, fibrocytes and smooth muscle cells [9,41,42]. An interesting study of Laukkanen’s group showed that thyroid CAFs may develop from resident thyroid mesenchymal stem cells (MSCs) upon paracrine interactions with cancer cells (Figure 2). They demonstrated that MSCs from PTCs are characterized by a higher expression of fibrotic markers, such as FAP, Col-1 and Tn-C, compared with MSCs from a normal thyroid gland. Consequently, the authors hypothesized that MSCs exposed to thyroid-cancer-cell-secreted factors acquire similar phenotypic features of CAFs and thus serve as an origin of cells for activated fibroblasts in the thyroid gland (Figure 2) [43].

In addition to FAPα, Tn-C and α-SMA proteins, other thyroid CAF markers can include vimentin, fibroblast specific protein (FSP), osteonectin and desmin, which are often used to identify CAFs from other solid tumors [9,21,44,45,46].

Reprogramming fibroblasts into CAFs also involves changes in their proliferative and migratory capabilities and in their synthetic and secretory functions (Figure 1). In vitro results demonstrated that fibroblasts treated with thyroid-tumor-cell-derived conditioned media differentiate into CAFs and increase their proliferation rate and migratory capability compared with non-treated normal fibroblasts [26,31]. Furthermore, CAFs differ from normal fibroblasts in their capability to synthesize and secrete ECM proteins and inflammatory proteins (Figure 1). It has been demonstrated that thyroid CAFs are the main source of type I collagen (Col-1), which is highly abundant in thyroid tumors but not in the normal thyroid gland. This evidence suggests a reduced capability of normal thyroid fibroblasts to produce Col-1 compared with thyroid CAFs [31]. Furthermore, it has been reported that human fibroblasts activated by thyroid-cancer-cell-secreted factors significantly enhance the expression and secretion of interleukin 6 (IL-6), which is an inflammatory cytokine involved in the progression of several types of cancer, including thyroid tumors [39,47,48]. This is consistent with other studies showing that CAFs, from several solid tumors, secrete higher levels of IL-6 than normal fibroblasts [49,50]. Another classic way to distinguish CAFs from normal fibroblasts involves analyzing their metabolic profiles. It is noteworthy that, during CAF differentiation, fibroblasts reprogram their metabolism and acquire a catabolic phenotype characterized by the inhibition of oxidative phosphorylation (OXPHOS) and activation of glycolytic pathways [34]. Fozzatti et al. demonstrated that soluble factors secreted by thyroid cancer cells not only enhance the state of activation of human fibroblasts but also trigger their metabolic switch toward a more glycolytic phenotype (Figure 1). In fact, after either co-culture with anaplastic cancer cells or treatment with tumor cell-derived conditioned medium, human fibroblasts significantly enhance the expression of both the glucose transporter GLUT-1 and the glycolytic enzyme lactate dehydrogenase A (LDH-A) [39].

Fibroblast growth factor (FGF) 21 is a hormone-like FGFs belonging to the FGF ligand family. The expression of FGF21 is significantly increased in response to various nutritional and metabolic alterations, such as mitochondrial dysfunction and endoplasmic reticulum stress [51]. FGF21 regulates glucose, lipid and energy metabolism. It has been demonstrated that high serum levels of FGF21 in PTC patients were significantly related to an aggressive tumor phenotype and poor prognosis. FGF21 increases the migratory and invasive capabilities of thyroid cancer cells via the induction of EMT. Furthermore, FGF21 promotes cancer progression via the upregulation and induction of the FGFR signaling axis, including AKT and ERK phosphorylation. It is noteworthy that targeting FGFR signaling reduced the pro-tumor effects of FGF21 on PTC cells. Therefore, FGF21 may be considered as a promising biomarker to predict cancer progression, and targeting FGFR may represent a new therapeutic approach for PTC patients with high FGF21 serum levels [51].

The study of pathways and molecules regulating CAF activation and differentiation in thyroid cancers is an emerging field in cancer research. However, little is currently known about these molecular mechanisms and further studies are needed. Fozzatti et al. identified the reactive species of oxygen (ROS), highly produced by ATC cells, as key molecules supporting CAF differentiation and CAF metabolic reprogramming in thyroid cancers (Figure 2) [39], as well as in other solid tumors [34]. They further speculated that fibroblast activation and differentiation into CAFs may be in part mediated by IL-6, which is an inflammatory cytokine highly secreted by ATC cells (Figure 2) [39]. In addition to ROS and IL-6, thyroid cancer cells secrete great amounts of platelet-derived growth factor (PDGF), which could serve as a causative molecule promoting fibroblast activation and CAF formation (Figure 2) [39]. Another signaling pathway promoting CAF differentiation involves the activation of the Src pathway and its downstream target Akt in fibroblasts [39]. It is well known that TGF-β sustains fibroblast activation and has pro-mitogenic and chemotactic effects on fibroblasts [52]. Accordingly, in human PTCs, a significant correlation was identified between a higher TGF-β1 expression in thyroid tumor cells and a higher α-SMA expression in fibroblasts (Figure 2) [53]. Therefore, TGF-β may be a potential regulator of CAF differentiation during thyroid cancer evolution, even if further investigations are required to verify its role.

## 4. Exploring the Role of Fibroblasts in Thyroid Cancers

Thyroid cancer is the most common endocrine malignancy and the contribution of thyroid cancer cells to cancer evolution has been widely investigated and analyzed. On the other hand, the contribution of the thyroid TME, especially of CAFs, to thyroid cancer development and progression remains largely underexplored. Fortunately, in the last years, several studies are slowly elucidating the specific role of thyroid CAF during stromal–cancer cell interaction and during thyroid cancer progression. Using an in vivo subcutaneous tumor model, Saitoh et al. [54] demonstrated that intrathyroidal stromal TME, particularly fibroblasts, can increase thyroid tumor cell growth. In particular, they demonstrated that tumorigenic Fisher rat thyroid follicular cells (FRTL-Tc) injected intrathyroidally grow faster than those injected subcutaneously, and that the co-injection of fibroblasts mixed with FRTL-Tc cells generates subcutaneous tumors larger than tumors derived from the injection of FRTL-Tc cells alone. This in vivo growth-promoting function of fibroblasts on FRTL-Tc cells was also confirmed by in vitro experiments demonstrating that soluble factors, present in a fibroblast-derived conditioned medium, promote the growth of FRTL-Tc cells [54]. Consistent with these results, Fozzatti et al. also demonstrated that soluble factors secreted in the conditioned medium of activated fibroblasts increase the in vitro proliferation and migration of FTC-133 cells (Figure 2). Furthermore, it has been reported that fibroblast-derived soluble factors increase the expression of vimentin, a marker of EMT, drive cytoskeletal re-organization and polarization and increase cancer cell protrusions, such as filopodia and lamellipodia (Figure 2) [39,55]. Although CAF-derived pro-tumorigenic factors remain underexplored in the thyroid tumor milieu, Fozzatti et al. speculated that IL-6 and ROS, highly produced by pro-tumorigenic CAFs, could represent key factors involved in thyroid cancer progression [39]. Furthermore, Parascandalo et al. demonstrated that the stromal superoxide dismutase 3 (SOD3), one of the main H_2_O_2_-producing enzymes [43,56], is highly produced by PTC MSCs, which might function as a source of myofibroblasts in thyroid cancers as previously mentioned. SOD3-derived PTC MSCs have been shown to support thyroid cancer cell proliferation and inhibit cancer cell migration [43]. This controversial role of SOD3 is in line with other studies showing the dual function of SOD3 in regulating cancer progression [56,57,58,59,60,61]. However, the authors speculated that an autocrine–paracrine conversion of SOD3 expression from cancer cells to the stromal compartment may justify the controversial role of SOD3 in cancer [43]. Specifically, they speculated that SOD3 bolsters the starting stage of tumorigenesis. Once SOD3 expression levels reach non-physiological toxic levels, cancer cells decrease the autocrine SOD3 generation. On the other hand, stromal cells increase SOD3 secretion. The stromal paracrine secretion of SOD3 then continues to support the proliferation of tumor cells, and, correspondingly, reduces the affinity of tumor cells to tumor stroma by decreasing the expression of chemotactic cytokines, such as IL1α and MCP-1, in the stromal compartment. For the authors, these mechanisms allow local intratumoral cancer cell migration and thus contribute to cancer progression [43]. Therefore, a better understanding of SOD3-coordinated signal transduction in tumor stroma and epithelial cancer cells should allow for the identification of new molecular targets and therapeutic strategies able to inhibit the tumor-supportive function of the stroma and thus hinder thyroid cancer progression. In addition, CAFs can synthesize and secrete CXCL12, which is a strong inflammatory factor, improving thyroid cancer cell proliferation, migration and invasion [62]. In particular, CXCL12 (also known as SDF-1) binds to CXCR4 and CXCR7 proteins on thyroid cancer cells and consequently activates multiple signaling pathways, including mTOR, ERK1/2, SAPK/JNK, Akt, p38 and BTK. It is known that ursolic acid (UA), an anti-inflammatory natural triterpenoid, decreases the secretion of CXCL12 by CAFs, thus exerting a strong anti-tumor activity in vitro. Interestingly, in addition to the indirect mechanisms through CAFs, UA was shown to inhibit thyroid cancer progression by reducing the expression levels of both CXCR4 and CXCR7 mRNA and protein [62]. This study, together with other preclinical and clinical evidence, demonstrated that targeting the CXCL12/CXCR4/CXCR7 axis could be a promising and powerful therapeutic strategy in thyroid cancers [62,63,64]

The pro-tumorigenic function of thyroid CAFs also reflects their ability to produce and remodel the thyroid ECM (Figure 2) [31,32]. In particular, CAFs, through a coordinated action with thyroid cancer cells, have been shown to enhance ECM stiffness through an aberrant and excessive deposition of Col-1 fibrils (Figure 2). In turn, thyroid cancer cells, through LOX expression, cross link fibroblast-derived collagen fibrils to generate mature and cross-linked collagen fibers. These collagen fibers delimit specific and privileged routes through which thyroid cancer cells can spread out from the primary tumor mass and reach local and distant secondary tumor sites [31]. LOX, as well as Col-1, have been identified as markers for human thyroid cancer aggressiveness [65] and predict poor overall survival in thyroid cancer patients [31]. Therefore, thyroid CAFs and their ability to remodel the ECM and generate a tumor-supportive niche can represent novel and promising therapeutic targets to hinder thyroid cancer progression. In vitro experiments of co-cultures revealed that the interaction between fibroblasts and thyroid cancer cells increases the secretion of pro-MMP-2 and pro-MMP-9 in the supernatants. Furthermore, a significantly higher prevalence of the active form of MMP-2 was observed in the supernatant obtained from fibroblast-thyroid cancer cell co-cultures compared with the levels of MMP-2 detected in the supernatant from fibroblast-non tumor thyroid cell co-cultures [55]. High expression levels of MMP-2 and MMP-9 are associated with an increased risk of developing lymph node metastasis in PTC patients [66]. Furthermore, PTC patients with high preoperative serum levels of MMP-2 are characterized by a larger tumor size, presence of lymph node metastasis, extrathyroidal invasion and an advanced TNM stage [67]. Long noncoding RNAs (lncRNAs) are increasingly investigated for their capability to affect the behaviors of both cancer and non-cancerous stromal cells. Regarding thyroid cancers, lncRNAs were shown to modulate cancer progression by influencing cancer cell growth, proliferation, invasion, metastases and differentiation. Of note, in PTCs, the lncRNA MEG3 was shown to be highly expressed in thyroid cancers, with lymph node metastases compared with tumors without lymph node metastases. Furthermore, a high MEG3 expression was highly associated with an increased CAF infiltration and MMP-2 expression in PTCs. Interestingly, the authors hypothesized that the expression of MEG3 in CAFs may enhance their capability to remodel the ECM through MMP-2 expression and thus favor the propensity for lymph node metastases. However, the correlation between MEG3 expression and MMP-2 production in CAFs requires necessary further investigation and validation in thyroid cancers [68].

It has been widely demonstrated that extracellular vesicles (EVs) released by both tumor cells and non-cancerous cells are important regulators of a great number of biological and cellular functions, including tumor–stroma interplay and tumor growth and progression [45,69,70]. Interestingly, by using an in vitro model of the thyroid TME, obtained through co-culture experiments, Donadio’s group demonstrated that EVs obtained from co-cultures of thyroid cancer cells and fibroblasts promote the secretion of pro-MMP-2 and the expression of the active form MMP-2 in normal fibroblasts. Furthermore, EVs from fibroblast–thyroid tumor cell co-cultures have also been characterized by an increased expression of the transmembrane glycoprotein CD147 [55], which is involved in the secretion and activation of MMPs [71]. CD147 was also shown to be highly expressed in thyroid tumor tissues, but not in normal tissues or nodular goiter. Moreover, in thyroid cancers, a positive correlation was found between the expression of CD147 and thyroid cancer progression, extrathyroidal invasion and lymph node metastasis [72,73,74]. Therefore, the study of Donadio and colleagues demonstrated, for the first time in the context of the thyroid tumor, that specialized EVs support thyroid cancer progression by mediating the crosstalk between tumor cells and stromal cells and by increasing the proteolytic activity of fibroblasts, which consequently enhance their protumor function [55].

Interestingly, a recent study demonstrated that thyroid CAFs facilitate tumor progression not only by affecting thyroid tumor cell behaviors but also by exerting immune-modulating functions (Figure 2). In thyroid tumors from PTC patients, CAFs were associated with an increase in monocytes and activated dentritic cells (DCs) and a decrease in M0 macrophages. Furthermore, it has been demonstrated that thyroid CAFs increase the expression of various immune checkpoints, such as CTLA4, PDL1/2 and IDO 1, and thus contribute to the immune escape of thyroid cancer cells and consequently to thyroid tumor growth (Figure 2) [30]. Additionally, a high CAF score in thyroid cancer patients was shown to be positively correlated with an increased expression of immune checkpoint markers, such as CD274, PDCD1LG2, CD86, CD80 and CTLA4, and an increased expression of markers of activated tumor-associated macrophages, including EMR1, CSF1R, CD163 and ITGM in DDTCs and PTCs. These results provide more important evidence of the involvement of thyroid CAFs in modulating immune cell functions (Figure 2) [33]. However, the current understanding of CAF-immune modulating functions in thyroid cancer is still primitive. Consequently, further studies are required to identify molecular signaling pathways regulating the CAF-immune modulating role in order to design potential novel anti-tumor therapeutic approaches able to abolish the pro-tumor immunity in thyroid cancer.

## 5. Therapeutic Perspectives

CAFs have been recognized for their pro-tumor action and genetic stability that make this stromal cell type an appealing target for novel therapeutic strategies targeting the TME and hindering tumor progression [41]. To the best of our knowledge, unfortunately, no therapies specifically targeting thyroid CAFs are currently approved or on clinical trial for thyroid cancer patients. However, accumulating evidence has demonstrated that the use of anti-fibrotic drugs [75] or molecules targeting CAF-derived pro-tumor factors, including TGF-β receptor-I/ALK5 inhibitors or TGF-β-neutralizing antibodies [76], inhibitors of IL-6 [77], recombinant fusion protein directed against FAP (such as RO6874281) [78] or nanovaccines targeting FAP [79], exerts a strong anti-tumor activity in several solid tumors [41]. CAF deactivation is another potent therapeutic approach able to enhance the current anti-tumor treatments. In particular, the induction of PEDF expression [80], the inhibition of the Notch signalling pathway [81] and the ablation of β-catenin [82] in CAFs were shown to suppress fibroblast activation, CAF differentiation and the CAF’s ability to promote cancer growth and progression. Furthermore, the presence of similarities between CAFs across different solid cancers suggests that discoveries made in one specific tumor type may have a wider effect on the whole field of oncology [83]. Hence, thyroid CAFs should also be considered as excellent and promising therapeutic targets in thyroid cancer. All of these observations strongly highlight the necessity to identify and develop novel therapies targeting thyroid CAFs to improve current anti-tumor treatments and thus enhance the outcome of thyroid cancer patients.

## 6. Conclusions

The heterogeneous fibroblast populations of TME is an emerging area of oncological research. In particular, it is known that CAFs can sustain thyroid cancer growth and progression by producing soluble factors and ECM proteins deeply influencing thyroid cancer cell behavior and aggressiveness. Moreover, CAFs also facilitate tumor progression through immune-modulating functions.

The CAF differentiation process and the interaction of these fibroblast populations with cancer cells could be possible targets for therapeutic strategies.

It is noteworthy that studies focusing on the possible reversibility of the CAF phenotype toward an inactivated and less pro-tumorigenic phenotype, restraining the growth and dissemination of thyroid cancer, could allow for a dramatic improvement in therapeutic strategies [46].

Finally, normal fibroblasts before their differentiation into CAFs can hinder cancer growth [45]. Hence, studies committed to the stabilization of the anti-tumor phenotype of normal fibroblasts could represent another innovative approach to inhibiting thyroid cancer development and progression [10].
